# A Study on the Attachment to Pets Among Owners of Cats and Dogs Using the Lexington Attachment to Pets Scale (LAPS) in the Basque Country

**DOI:** 10.3390/ani15010076

**Published:** 2025-01-01

**Authors:** Eider Egaña-Marcos, Olatz Goñi-Balentziaga, Garikoiz Azkona

**Affiliations:** 1 Department of Basic Psychological Processes and Their Development, Euskal Herriko Unibertsitatea (UPV/EHU), Tolosa Hiribidea, 20018 Donostia, Spain; eganaeider1@gmail.com; 2 Department of Clinical and Health Psychology and Research Methodology, Euskal Herriko Unibertsitatea (UPV/EHU), Tolosa Hiribidea, 20018 Donostia, Spain; olatz.goni@ehu.eus

**Keywords:** attachment, human–pet interaction, cat owner, dog owner, Lexington attachment to pets scale

## Abstract

The relationship between humans and their pets has long fascinated researchers, particularly in exploring how attachment varies according to the type of pet. Cats and dogs exhibit unique behavioral and social traits that influence the dynamics of human–pet relationships. Moreover, specific human characteristics have been found to affect this attachment. Our study examines the human factors that influence pet attachment among cat and dog owners in the Basque Country, located in northern Spain. By investigating these elements, our research aims to enhance the understanding of how human factors shape the human–animal bond. The study included a total of 202 participants, of whom 66.8% were dog owners, and 74.8% identified as women, with ages ranging from 18 to 74 years. Consistent with many previous studies, our results indicate that attachment is generally stronger with dogs compared to cats and that owner’s characteristics such as being female, younger, not living with children, and the amount of time spent with pets on weekends are linked to stronger attachments to pets.

## Simple Summary

The bond between humans and their pets has long captivated researchers, particularly in understanding how attachment varies based on the type of pet. Cats and dogs display distinct behavioral and social characteristics that shape the dynamics of human–pet relationships. Furthermore, certain human traits have also been identified as influencing this attachment. Our study investigates factors affecting pet attachment among cat and dog owners in the Basque Country, located in northern Spain. By investigating these aspects, our research aims to confirm the human factors that influence the human–animal bond in a previously unstudied population. Our findings confirm that attachment tends to be notably stronger with dogs than with cats and that owner’s traits such as being female, younger, not living with children, and the amount of time spent with pets on weekends are associated with stronger attachments to pets.

## 1. Introduction

Through Law 7/2023 on the Protection of Animal Rights and Welfare, the Spanish legal system defines pets as “domestic or wild animals in captivity, kept by humans, primarily in the home” [1]. A recent worldwide survey indicated that 58% of respondents owned pets. Of these pet owners, 59% had dogs and 53% had cats [2]. In Spain, dogs and cats are the preferred companion animals [3]. In fact, there are 10,165,498 dogs and 967,834 cats registered [4], meaning there are more pets than people under 18 (8,589,495 out of a total population of 48,592,909) [5]. The same trend is reported in the Basque Country, a northern region where 392,234 dogs and 32,138 cats are registered [4] and the under-18 population is 399,011 (total population of 2,196,745) [6].

Global pet ownership is estimated to grow, particularly in millennial households (1980–1994), which tend to have smaller families and children later in life [7]. Economic factors also play a role; while pets require some investment, their maintenance costs are generally lower [8]. In any case, pet owners value their relationship with their pet very highly, as shown by an international survey of dog and cat owners in which 95% of respondents considered their pet to be a member of the family and almost 90% described their relationship as close [9].

The human–animal interaction that results in a human–animal bond is a critical factor in the existence of multi-species families. The human–animal bond has been described as a mutually beneficial relationship between humans and animals. This bond is influenced by behaviors that are essential for the mental, physical, and social health and well-being of both parties [10]. Whereas few studies have focused on the benefits for the animals [11,12,13], numerous studies have shown how beneficial interactions with pets can be for human health, particularly in regard to stress management [14,15,16,17,18,19]. One of the main explanations for how people benefit from interacting with their pets is the social support they provide [20,21]. A recent systematic review showed that there are no discernible differences between dogs and cats in their relationships with their owners and pet owners’ perceptions of loneliness and social isolation. This suggests that both species provide companionship and emotional support [22].

Several theories attempt to explain the foundations of the human–animal bond. One key explanation is the biophilia theory, which suggests that humans have a natural interest in nature and, by extension, in animals. [23,24]. Although the domestication of dogs predates that of cats [25,26], both species have long coexisted in our society. Historically, the relationship between dogs and humans has been more cooperative, involving elements such as assisting with hunting. However, while cats have provided a service to humans in the sense that they hunt mice, their relationship with humans has always been less cooperative [27].

A second model posits that the bond between humans and animals is based on attachment [28,29], a universal human trait in affiliative behavior [30,31]. This suggests that humans have an innate need to connect with others, forming bonds of affiliation and affection. This need for connection may also explain why attachments can form with individuals of other species [24,32,33].

The bond between humans and their pets has long been a topic of fascination for researchers, with a particular focus on how attachment differs depending on the type of pet [34,35,36]. Certain behaviors of dogs and cats toward humans are similar, whereas others are different. The ability of both species to follow human pointing is similar, but dogs are more likely to look at their owners when faced with a problem-solving scenario in which they need their owners’ assistance to obtain food [37]. Dogs are generally perceived as more interactive, trainable, and dependent on human attention. Dogs tend to be more social and responsive to human interaction, exhibiting bonding behaviors such as tail wagging, eye contact, and playfulness. These behaviors often lead to stronger bonds, as dogs are perceived as more interactive and affectionate companions [38]. In contrast, cats are often perceived as more autonomous and self-reliant, characteristics that may lead to a different type of attachment, one that is less intense but still meaningful [39], which may contribute to a relationship that is perceived as more “coexistent” than “companionate” [40]. Cat owners tend to place a high value on their cats’ autonomy, a trait that may be less desirable in dogs [41,42]. From this perspective, it is possible that the traditional gap in the amount of care owners provide for their cats may be due to a natural human response to the different behavioral characteristics of cats and dogs [43].

In human bonding processes, eye contact is fundamental in establishing closeness and expressing feelings of love and loyalty, and, at the neuroendocrine level, oxytocin plays an important role in bonding [44,45]. Recently, it has been described that oxytocin is released in both dogs and owners when they look at each other, suggesting that humans feel an affection for their dogs similar to that felt for family members [46]. It has also been shown that interbrain synchronization occurs within an interacting human–dog dyad, which may be responsible for interspecies communication [47]. In cats, one study found that intranasally administered oxytocin increased male, but not female, gaze toward humans [48]. The male-specific increase in gaze toward humans observed in this study differs from previous research on dogs, where such effects were observed only in females [46]. These findings suggest that exogenous oxytocin may have a general effect on cats’ social relationships with humans as well as the possibility of different mechanisms between cat–human and dog–human relationships.

The Lexington Pet Attachment Scale (LAPS) is one of the most popular scaled instruments used to measure the quality of attachment between pet owners and their pets. It has been applied to many species, although it is primarily aimed at cat and dog owners, and has been used in various countries, including Australia [49], Austria [50], Brazil [51], Canada [52], Denmark [50], France [53], German [54], Italy [13,55,56,57], Mexico [58,59], New Zealand [49], Portugal [60], the United Kingdom (UK) [49,50], and the United States of America (USA) [35,61,62,63,64,65,66]. These studies have shown that dog owners generally score higher than cat owners and that the demographic characteristics of the owner, such as age, gender, and education, for example, influence the level of attachment. Interestingly, one study found that dog–human attachment was only influenced by owner characteristics [13].

We are aware of only one study that analyzed attachment using the short version of the LAPS in Spain. This study found that mean scores were very high among volunteers from shelters in Andalusia, a southern region [67]. Given the lack of data in Spain, this work aimed to explore the level of attachment between cat and dog owners in the Basque Country, a region in the north of the country, and the effect of pet owners’ demographic factors on pet attachment. By examining these aspects, our research aims to confirm the human factors that influence the human–animal bond in a previously unstudied population.

## 2. Materials and Methods

### 2.1. Participants and Procedure

The participants were recruited online between 10 July 2023 and 30 September 2024 through email lists provided by various Basque veterinary colleges, where veterinarians were asked to disseminate the invitation among their clients. Additionally, a snowball sampling technique was employed: respondents were asked to invite other cat or dog owners to participate in the survey. The study was restricted to current cats or dogs owners living in the Basque Country (Spain) who were over 18 years old. Participants who owned both cats and dogs simultaneously were excluded.

In a cover letter attached to the questionnaire, participants were informed that the survey data would be used for scientific purposes only and that their responses would remain completely anonymous. All participants provided voluntary informed consent before completing the short online questionnaire, which took approximately 10 min (via the Google Drive platform). The study was conducted in accordance with the guidelines established by the Declaration of Helsinki. All procedures and informed consent protocols were approved by the Ethics Committee for Human-Related Research (CEISH) of the University of the Basque Country (UPV/EHU); M10/2023/222.

### 2.2. Instruments

The survey included questions about participants’ personal information, such as whether they were cat or dog owners, gender, sexual orientation, age (years), education, sentimental relationship (*yes*/*no*), household composition, area of residence, salary range, number of pets they lived with, who usually took care of the pets, how long they had lived with their pets (in years), and how much time (in hours) they spent with their pets during the weekdays and on weekends.

The attachment to cats and dogs was assessed using the Spanish version of the LAPS [59], substituting the term dog (“*perro*”) for pet (“*animal de compañia*”). This scale comprises 23 items rated on a four-point Likert-type scale (0 = *strongly disagree*; 3 = *strongly agree*) and measures three subscales: General Attachment (GA), Person Substitution (PS) and Animal Rights (AR). The GA subscale reflects the level of affection between the owner and the pet, whereas the PS subscale measures how central the pet is in the owner’s life. Finally, the AR subscale indicates the pet’s role or status within the household. The participants were asked to answer the questions with their current pet(s) in mind. Supplementary Table S1 shows the means and standard deviation of the 23 items. The reliability analysis demonstrated very good internal consistency for all tests (α = 0.89, ω = 0.91).

### 2.3. Statistical Data Analysis

All statistical analyses were performed using the Jamovi software package (version 2.3.21.0) and GraphPad Prism software package (version 10.3.1), with the significance level set to *p* < 0.05. Frequency (%) and distribution statistics (mean ± standard deviation (SD), median, and minimum-maximum) were used to describe the sample. Mean, standard deviation, and homogeneity indices were calculated for each LAPS item. The reliability of the LAPS total score and subscales was analyzed using standardized Cronbach’s alpha (α) and McDonald’s ordinal omega (ω) coefficients.

The normality test (Shapiro–Wilk) indicated a non-parametric distribution for all variable scores. Subsequently, Mann–Whitney U tests (for variables with two categories) or Kruskal–Wallis one-way analyses of variance (for variables with more than two categories) were conducted to analyze differences in LAPS scores. To calculate effect sizes, we used the rank biserial correlation (rrb), reference values of <0.3 (small effect), 0.3–0.5 (moderate effect), and >0.5 (large effect), and the Squared Epsilon coefficient (ε^2^), reference values of 0.01–<0.06 (small effect), 0.06–<0.14 (moderate effect), and ≥0.14 (large effect). We used a two-way ANOVA to analyze the possible interaction of species with gender and with the factor of living with children at home, and specific comparisons were analyzed using the post hoc Tukey test. A Cohen’s d (d) test for the effect size was performed to estimate the strength of the effects between two groups (>0.8 large effect, 0.5–0.8 moderate effect, and < 0.5 small effect).

Associations between parameters were analyzed using a bivariate Spearman correlation (rho) with interpretations of <0.09 (very small effect), 0.10–0.29 (small effect), 0.30–0.49 (moderate effect), and >0.50 (large effect). There were no missing data. Only significant differences between groups are presented in the results section; small effect sizes are not considered.

From this initial analysis, we observed that certain factors (species, gender, age and living with/without children) could influence the total LAPS score and its subscales. Time spent with pets at weekends could influence the LAPS, and time spent during the weekdays and at weekends (also total time) could influence the GA and AR scores. These factors met the criteria for statistical significance in the analysis of variance and were correlated. To determine the independent influence of these variables, we then performed linear regression analyses.

## 3. Results

### 3.1. Participants’ Information

A total of 202 individuals completed the survey (Table 1), of whom more than half were dog owners (66.8%). The vast majority identified as women (74.8%) and heterosexual (80.2%). Their ages ranged from 18 to 74 years (mean: 39.1 ± 13.8; median: 38). Slightly more than half of the participants had a university degree (63.8%) and no romantic partner (59.4%). Most participants lived accompanied at home (83.7%), without children (71%), and in urban areas (82.7%). The majority of participants earned between EUR 12,000 and EUR 52,000 per year (68.8%). Slightly less than half of the participants lived with one pet (69.3%) and shared its care (53.5%). The median number of close friends was 5 (0–40), and the median number of close relatives was three (0–17), bringing the total median number of people in the participants’ most intimate circle to eight (1–55). There were no significant differences in the number of close friends and relatives according to species.

Cat owners reported living with their pets for significantly longer periods of time than dog owners (U = 3561.5, *p* = 0.014, rrb = 0.21). Cat owners had been living with them for an average of 6 ± 4.8 years (median: 4, ranging from 4 months to 18 years), whereas dog owners reported an average of 7.2 ± 4.2 years (median: 7, ranging from 2 months to 19 years). Both groups of pet owners reported spending similar amounts of time with their pets during weekdays (cat owners: 49.6 ± 39.2 h, median: 40, ranging from 1 to 120 h; dog owners: 49.8 ± 40.5 h, median: 35, ranging from 1 to 120 h). However, cat owners reported spending slightly less time than dog owners on weekends (21.1 ± 14.4 h, median: 18, ranging from 1 to 48 h, vs. 26.7 ± 15.9 h, median: 24, ranging from 1 to 48 h, respectively) (U = 3615, *p* = 0.02, rrb = 0.20). Overall, cat owners spent 70.7 ± 50.1 h per week interacting with their cats (median: 62, ranging from 2 to 168 h), whereas dog owners spent 76.6 ± 52.9 h per week (median: 64, ranging from 2 to 168 h).

### 3.2. Attachment to Pets

Cat owners scored lower on the total LAPS (U = 3060, *p* < 0.001, rrb = 0.32) and on the Person Substitution subscale (U = 2871, *p* < 0.001, rrb = 0.36) than dog owners (Table 2). Significant differences with small effect sizes were observed in the General Attachment (U = 3399, *p* = 0.004, rrb = 0.24) and Animal Rights (U = 3665, *p* = 0.027, rrb = 0.19) subscales.

Significant differences were found according to the gender of the participants and according to whether or not they were living at home with children. No statistical differences were observed for the other variables described in Table 1. Participants who identified as female scored higher on the LAPS (U = 2619.5, *p* < 0.001, rrb = 0.32) and on the Person Substitution subscale (U = 2556, *p* < 0.001, rrb = 0.34) than those who identified as male. Gender-significant differences were also observed on the General Attachment (U = 2913, *p* = 0.009, rrb = 0.24) and Animal Rights (U = 3046, *p* = 0.024, rrb = 0.21) subscales, though with small effect sizes (Table 3).

**Figure 1:**
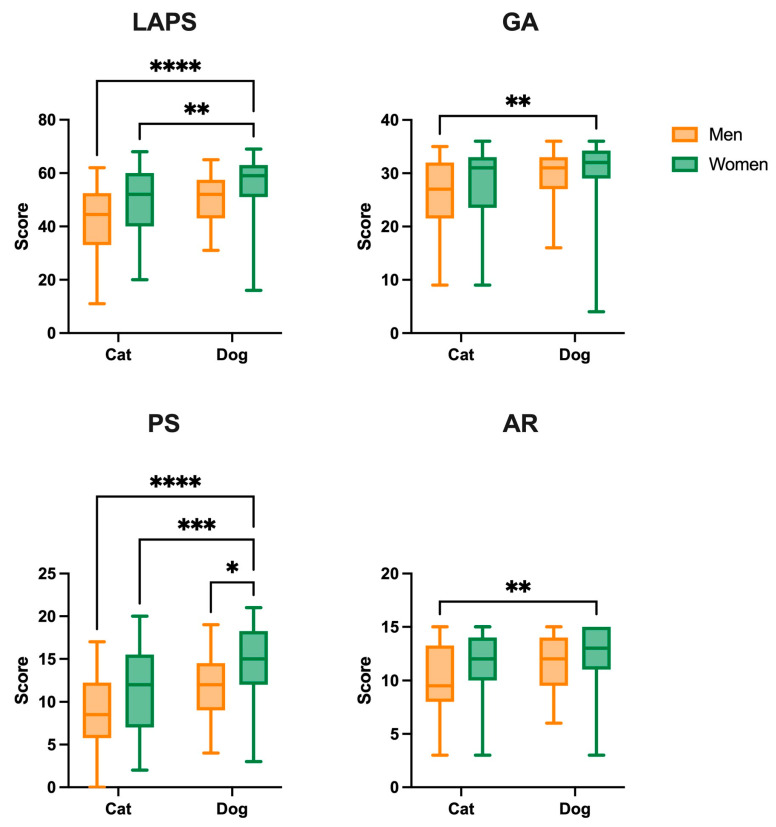
Lexington Attachment to Pets Scale (LAPS), General Attachment (GA), Person Substitution (PS), and Animal Rights (AR) results by gender and species. Data are presented as group median (min to max). * *p* < 0.05, ** *p* < 0.01, *** *p* < 0.001 and **** *p* < 0.0001.

We analyzed whether gender influenced attachment to cats or dogs using a two-way ANOVA (gender and species) analysis. Statistical analysis revealed no gender–species interaction (Figure 1) but showed significant differences for the species factor in the LPAS total score (F _(1,198)_ = 13.69, *p* = 0.0003) and the GA (F _(1,198)_ = 8.935, *p* = 0.0032), PS (F _(1,198)_ = 16.20, *p* < 0.0001), and AR (F _(1,198)_ = 5.822, *p* = 0.017) subscales, as well as for the gender factor in the LPAS (F _(1,198)_ = 8.553, *p* = 0.0039) and the PS (F _(1,198)_ = 11.74, *p* = 0.0007) and AR (F _(1,198)_ = 5.581, *p* = 0.0191) subscales. The results showed that female dog owners scored higher on all scales (Supplementary Table S2). Female dog owners scored significantly higher than male cat owners (*p* < 0.0001, d = −1.13) and female cat owners (*p* = 0.007, d = −0.57) in regard to total LPS. Female dog owners also scored significantly higher in PS compared to male cat owners (*p* < 0.0001, d = −1.27), female cat owners (*p* < 0.001, d = −0.70), and male dog owners (*p* = 0.017, d = −0.59). Additionally, female dog owners scored significantly higher than male cat owners in GA (*p* = 0.008, d = −0.82) and AR (*p* = 0.009, d = −0.81).

Participants who lived with children were found to score lower on the LAPS (U = 1955, *p* < 0.001, rrb = 0.34) and the General Attachment (U = 2210, *p* = 0.011, rrb = 0.25), Person Substitution (U = 1951, *p* < 0.001, rrb = 0.34), and Animal Rights (U = 1933, *p* < 0.001, rrb = 0.34) subscales compared to those who did not live with children (Table 4).

**Figure 2:**
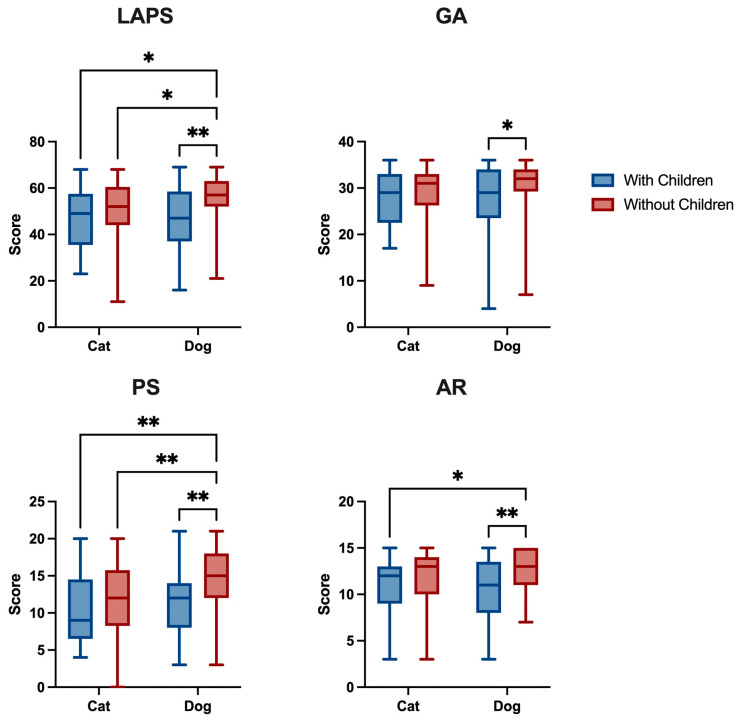
Lexington Attachment to Pets Scale (LAPS), General Attachment (GA), Person Substitution (PS) and Animal Rights (AR) results by living with or without children and species. Data are presented as the group median (min to max). * *p* < 0.05, ** *p* < 0.01.

A two-way ANOVA (children and species) analysis revealed no children–species interactions (Figure 2), but there were significant differences in the children factor in the LPAS total score (F _(1,166)_ = 8.393, *p* = 0.0043) as well as the GA (F _(1,166)_ = 5.730, *p* = 0.0178), PS (F _(1,166)_ = 6.679, *p* = 0.0106) and AR (F _(1,166)_ = 10.74, *p* = 0.0013) subscales, although there were only differences in the species factor in the PS subscale (F _(1,166)_ = 6.246, *p* = 0.0134). Dog owners living without children at home scored higher on all scales (Supplementary Table S3). Dog owners without children at home scored significantly higher in the total LAPS than cat owners with children (*p* = 0.037, d = −0.54) and without children (*p* = 0.017, d = 0.98) and dog owners with children (*p* = 0.007, d = 0.70). A similar trend was observed in PS, where dog owners without children scored significantly higher than cat owners with children (*p* < 0.01, d = 1.02) and without children (*p* < 0.01, d = −0.65) and dog owners with children (*p* < 0.01, d = 0.61). Additionally, dog owners without children scored significantly higher in GA compared to dog owners with children (*p* = 0.038, d = 0.57). They also scored higher in AR compared to cat owners (*p* = 0.011, d = 0.79) and dog owners (*p* = 0.003, d = 0.74) living with children.

### 3.3. Bivariate Correlation Analyisis

A correlational analysis revealed a weak negative correlation between age and the LAPS total score (−0.26, *p* < 0.001), as well as the General Attachment (−0.22, *p* = 0.01), Person Substitution (−0.29, *p* < 0.001) and Animal Rights (−0.25, *p* < 0.001) subscales. A weak positive correlation was observed between time spent on weekdays and the General Attachment (0.16, *p* = 0.02) and Animal Rights (0.15, *p* = 0.04) subscales. A weak positive correlation was also observed between time spent on weekends and the LAPS (0.20, *p* = 0.005), as well as the General Attachment (0.24, *p* < 0.001) and Animal Rights (0.15, *p* = 0.03) subscales.

### 3.4. Linear Regression

Previous analyses have shown that certain qualitative (species, gender, living with or without children at home) and quantitative (age and time spent with pets) variables influence LAPS scores and the GA, PS and AR subscales. To determine the influence of each of these variables, we performed a regression analysis.

The linear regression model revealed that approximately 24.7% of the variability in total LAPS is explained by the independent variables, while the amount is 14.9% for GA, 23.3% for PS, and 16.1% for AR. Among the variables, species was found to have a significant effect in the LAPS and the PS subscale, gender in the LAPS and PS subscale, age in the LAPS and the PS and AR subscales, and living with/without children in the LAPS and the GA and AR subscales. Time during the weekend was only significant in the LAPS regression model (Table 5).

## 4. Discussion

Many human characteristics have been identified as influencing the strength and nature of the human–animal bond. In our study, we investigated the relationship between the strength of the human–animal bond and the characteristics of cat and dog owners. We included only owners of either cats or dogs, but not both, to ensure that the bond measurement was species-specific and in order to minimize the possible bias arising from owning several pets of different species.

In our study, we first analyzed the psychometric characteristics of the adaptations we made for the Mexican version of the LAPS to generalize it to different pet species within the Spanish population (including Spanish speakers from other countries). As mentioned in the introduction, there is only one study in Spain that explores pet attachment, and it was conducted in the south of the country with volunteers from shelters rather than with pet owners. Although our study focused on a region in the north of the country, mainly for reasons of sample access, we believe that our results can be extrapolated to the rest of the country.

Our findings, along with previous research [49,50,54,61,65,66], support the notion that dog owners tend to have higher levels of attachment, consistently scoring higher on the LAPS than cat owners worldwide. When comparing LAPS scores across different studies, we found that not all of them use the same numerical scale. Although the original work by Johnson et al. (1992) [61] used a scale from 0 to 3, other studies have evaluated the scale from 1 to 4 [41,68,69,70] or even 1 to 5 [60,67]. Therefore, to accurately compare the total LAPS scores between our study and others, we included only those studies that used the original numerical scale, which is also used in our study.

Overall, the average LAPS values for cat and dog owners in our study were similar to those in studies from Austria [50], New Zealand [49], and the UK [50]; slightly lower than those from Brazil [51], France [53] and Germany [54]; and slightly higher than those from Denmark [50] and the USA [61,71,72]. A similar trend was observed when comparing the results of cat and dog owners separately. In a study conducted exclusively among dog owners in Italy [55], the total LAPS scores were slightly higher than ours, whereas, in Mexico [59], the scores were slightly lower. However, based on the categorization proposed by Marinelli et al. (2017)—with medium (23–46) and high (47–69) categories [13]—our results would fall within the upper part of this categorization. Consistent with these findings, a recent study of dog and cat owners from various parts of the world, mostly from the Global North [73], found no significant differences based on geographic location, and the results were very similar to ours. These data suggest a cultural globalization in the relationship patterns between cat and dog owners in Northern countries.

Our findings are consistent with previous research on owner characteristics that influence the strength of the pet–owner bond. Women scored higher on the LAPS, a result that aligns with all prior studies [49,50,51,53,54,56,57,61,73,74,75]. Specifically, we observed that female owners of dogs reported the highest attachment levels. This gender difference may stem from different social and cultural expectations, with women often being encouraged to display nurturing behaviors [76], which can lead to higher attachment scores. Additionally, women are generally more inclined than men to engage in positive behaviors and hold favorable attitudes toward animals, such as viewing pets as family members, supporting animal welfare, and opposing their exploitation [77]. These factors contribute to a stronger emotional bond with animals.

In the original 1992 study, older adults (aged 60 and above) had higher scores on the LAPS [61], and two subsequent studies found no significant relationship between an owner’s age and their attachment to pets [13,49]. However, our findings show that younger participants scored higher in regard to pet attachment than older participants—a pattern also observed in recent studies [54,56,73]. This shift may reflect changes in the role of pets among younger generations that are influenced by evolving cultural norms, increased awareness of animal welfare, and shifting social dynamics. This trend is particularly notable in Western cultures, where pets are increasingly anthropomorphized and valued as close companions. Pets are often given human names, showcased on social media, and viewed as family members rather than property [78,79].

Another factor that influences attachment to pets is the presence of children in the household [53,54,61]. Our data indicated that participants living with children scored lower on the LAPS, a trend previously observed among dog owners [13], though not among cat owners [80]. This distinction between dog and cat owners was also evident in our study. In this context, individuals without children may view pets as substitutes for offspring, thereby fostering stronger emotional connections. Prior studies support this idea, suggesting that voluntarily childless women may perceive their attachment to pets as comparable to, or even exceeding, the bonds typically formed between parents and their children [81]. Overall, these findings imply that pets play a distinctive role in filling familial voids, particularly in the absence of children or within certain family structures.

The time an owner spends with their pet is another factor influencing owner–pet attachment. This result appears to be species-influenced, as our data show that dog owners spent more time with their pet(s) during the weekend than cat owners. Generally, the more time spent with a pet, the stronger the attachment, as frequent interactions help to strengthen the bond [82]. Dog owners, for instance, often spend more time actively engaging with their pets due to the need for regular walks and outdoor activities, which may contribute to the higher attachment levels typically seen in dog owners compared to cat owners [60]. In contrast, cat owners might engage in fewer direct interactions, as cats are generally more independent and do not require as much attention or structured activity as dogs. Thus, certain species-specific interactions can strengthen pet–owner attachments. Research has shown that pet owners who regularly engage in caregiving activities—such as feeding, grooming, or playing—tend to report stronger attachment levels [13]. However, the quality of interactions also plays an important role; meaningful interactions that foster companionship and mutual understanding can enhance the human–animal bond regardless of the total time spent together.

Our study, however, did not identify certain owner characteristics—such as educational level [53,54,61], residential area [66,83,84], income range [53,54,61], relationship status [61,66], or social network size [61]—as predictors, contrary to findings from previous research. Differences in demographic factors, such as the cultural background of the participants, may have contributed to these outcomes. Additionally, discrepancies may be due to variations in sample size and recruitment methods, which can influence both the generalizability and reliability of the findings. Further studies using more diverse and representative samples are necessary to better understand the role of these variables and to determine whether our results reflect broader patterns or are specific to our sample.

Our study has several potential limitations. First, there are concerns regarding the use of the animal welfare questions in the LAPS, particularly the fact that some questions are framed negatively whereas others are framed positively. Moreover, the LAPS focuses on the emotional aspect of the pet–owner relationship, without accounting for other factors that may also be important, such as the personality of the owners. Research has shown that attachment levels can be linked to specific personality traits [71,73,85]. Second, the reliance on self-reported questionnaires introduces the potential for response bias, as it depends on participants’ honesty. Third, our study had a predominance of female participants, a trend also seen in most of the previous studies we referenced. This may reflect the fact that women are generally more likely to respond to online surveys [86]. Future research should aim to increase male representation for a more balanced perspective.

## 5. Conclusions

Our findings support the conclusion that attachment is generally more pronounced with dogs compared to cats and that certain owner characteristics—such as being female, younger, not living with children, and the amount of time spent with pets on weekends—are associated with stronger bonds with pets.

## Figures and Tables

**Table 1:** Participants’ personal information.

	Cat Owner	Dog Owner	General
	n (%)		
	67 (33.2%)	135 (66.8%)	202 (100%)
**Gender**			
Female	49 (73.1%)	102 (75.5%)	151 (74.8%)
Male	18 (26.8%)	33 (24.5%)	51 (25.2%)
**Sexual orientation**			
Bisexual	13 (19.4%)	11 (8.1%)	24 (11.9%)
Heterosexual	51 (76.2%)	111 (82.2%)	162 (80.2%)
Homosexual	3 (4.4%)	13 (9.7%)	16 (7.9%)
**Education**			
Primary school	4 (5.9%)	2 (1.5%)	6 (2.9%)
Secondary school	8 (11.9%)	15 (11.1%)	23 (11.3%)
Vocational training	11 (16.4%)	21 (15.5%)	32 (15.8%)
Undergraduate degree	41 (61.1%)	88 (65.2%)	129 (63.8%)
Ph.D.	3 (4.5%)	9 (6.7%)	12 (5.9%)
**Sentimental relationship**			
Yes	25 (37.3%)	57 (42.2%)	82 (40.6%)
No	42 (62.7%)	78 (57.8%)	120 (59.4%)
**Household composition**			
Live alone	12 (17.9%)	21 (15.5%)	33 (16.3%)
Live accompanied	55 (82.1%)	114 (84.5%)	169 (83.7%)
Without children	36 (65.5%)	84 (73.7%)	120 (71%)
With children	19 (34.5%)	30 (26.3%)	49 (29%)
**Living area**			
Rural	9 (13.4%)	26 (19.3%)	35 (17.3%)
Urban	58 (86.6%)	109 (80.7%)	167 (82.7%)
**Salary range** (euros/year)			
Prefer not to say	9 (13.5%)	25 (18.5%)	34 (16.8%)
<12,000	8 (11.9%)	14 (10.3%)	22 (10.9%)
12,000–<28,000	30 (44.8%)	44 (32.6%)	74 (36.6%)
28,000–<52,000	19 (28.3%)	46 (34.2%)	65 (32.2%)
≥52,000	1 (1.5%)	6 (4.4%)	7 (3.5%)
**Number of pets**			
One	39 (58.2%)	101 (74.8%)	140 (69.3%)
Two	25 (37.3%)	26 (19.2%)	51 (25.2%)
Three	1 (1.5%)	3 (2.2%)	4 (1.9%)
Four	0	4 (2.9%)	4 (1.9%)
Five	1 (1.5%)	1 (0.7%)	2 (0.9%)
Six	1 (1.5%)	0	1 (0.5%)
**Who takes care**			
Myself	33 (49.2%)	47 (34.8%)	80 (39.5%)
Shared responsibility	31 (46.3%)	77 (57.1%)	108 (53.5%)
Usually someone else	3 (4.5%)	11 (8.1%)	14 (7%)

**Table 2:** Lexington Attachment to Pets Scale (LAPS) results by pet owners. * *p* < 0.05, ** *p* < 0.01, *** *p* < 0.001.

		Mean	SD	Median	Range
**LAPS Total score**					
	General	51.8	12.02	54	11–69
	Cat owners	47.1 ***	13.28	50	11–68
	Dog owners	54.1	10.66	56	16–69
**General Attachment**					
	General	29.5	6.24	31	4–36
	Cat owners	27.6 **	7.07	29	9–36
	Dog owners	30.5	5.57	32	4–36
**Person Substitution**					
	General	12.9	4.97	13	0–21
	Cat owners	10.8 ***	5.02	10	0–20
	Dog owners	14.0	4.59	14	3–21
**Animal Rights**					
	General	12.0	2.78	13	3–15
	Cat owners	11.3 *	3.12	12	3–15
	Dog owners	12.3	2.55	13	3–15

**Table 3:** Lexington Attachment to Pets Scale (LAPS) results by gender. * *p* < 0.05, ** *p* < 0.01, *** *p* < 0.001.

		Mean	SD	Median	Range
**LAPS Total score**					
	Men	47.6	11.21	49	11–65
	Women	53.2 ***	11.98	55	16–69
**General Attachment**					
	Men	28.2	5.87	29	9–36
	Women	30.0 **	6.32	32	4–36
**Person Substitution**					
	Men	10.8	4.38	11	0–19
	Women	13.6 ***	4.97	14	2–21
**Animal Rights**					
	Men	11.2	3.00	12	3–15
	Women	12.3 *	2.67	13	3–15

**Table 4:** Lexington Attachment to Pets Scale (LAPS) results by living or not with children at home. * *p* < 0.05, *** *p* < 0.001.

		Mean	SD	Median	Range
**LAPS Total score**					
	Living with children	46.4	13.5	47	16–69
	Living without children	53.9 ***	10.9	55.5	11–69
**General Attachment**					
	Living with children	27.2	7.6	29	4–36
	Living without children	30.5 *	5.5	32	7–36
**Person Substitution**					
	Living with children	11	4.7	10	3–21
	Living without children	13.6 ***	4.7	14	0–21
**Animal Rights**					
	Living with children	10.7	3.2	11	3–15
	Living without children	12.5 ***	2.4	13	3–15

**Table 5:** Results of the linear regression analysis for LAPS involving predictor variables of pet species and participants gender, age, living with or without children, and time spent with their pet(s). * *p* < 0.05, ** *p* < 0.01, *** *p* < 0.001. Reference level: cat, male, no children.

		Standardized β	95% CI	t	*p*
**LAPS Total score**					
	R^2^ = 0.247; _adj_R^2^ = 0.224; F_(5, 163)_ = 10.74945, *p* < 0.001				
	Species	0.353	0.06–0.64	2.41	0.017 *
	Gender	0.338	0.016–0.66	2.08	0.039 *
	Age	−0.253	−0.41–−0.09	−3.12	0.002 **
	Children	−0.419	−0.76–−0.07	−2.41	0.017 *
	Time during weekend	0.247	0.11–0.39	3.46	<0.001 ***
**General Attachment**					
	R^2^ = 0.179; _adj_R^2^ = 0.149; F_(6, 162)_ = 5.92587, *p* < 0.001				
	Species	0.264	−0.04–0.57	1.68	0.091
	Gender	0.179	−0.16–0.52	1.03	0.302
	Age	−0.162	−0.33–0.01	−1.98	0.059
	Children	−0.409	−0.76–−0.04	−2.24	0.028 *
	Time during weekdays	0.119	−0.07–0.31	1.22	0.224
	Time during weekend	0.193	−0.001–0.39	1.88	0.061
**Person Substitution**					
	R^2^ = 0.251; _adj_R^2^ = 0.233; F_(4, 164)_ = 13.76174, *p* < 0.001				
	Species	0.553	0.27–0.83	3.83	<0.001 ***
	Gender	0.529	0.21–0.84	3.31	0.001 **
	Age	−0.251	−0.41–−0.09	−3.20	0.002 *
	Children	−0.309	−0.65–0.03	−1.789	0.075
**Animal Rights**					
	R^2^ = 0.191; _adj_R^2^ = 0.161; F_(6, 162)_ = 6.3890, *p* < 0.001				
	Species	0.163	−0.14–0.47	1.05	0.293
	Gender	0.357	0.02–0.69	2.07	0.039 *
	Age	−0.208	−0.37–−0.04	−2.47	0.025 *
	Children	−0.497	−0.85–−0.14	−2.75	0.007 **
	Time during weekdays	0.096	−0.09–0.28	0.98	0.326
	Time during weekend	0.112	−0.08–0.31	1.09	0.275

## Data Availability

The study data will be made available upon reasonable request to the corresponding author.

## Supplementary Materials

The following supporting information can be downloaded at: https://www.mdpi.com/article/10.3390/ani15010076/s1, Table S1: Lexington Attachment to Pets Scale: Spanish Version; Table S2: Lexington Attachment to Pets Scale (LAPS) results by pet owners and gender; Table S3: Lexington Attachment to Pets Scale (LAPS) results by pet owners and children presence at home.
