# Supplementary material for: A Study on the Attachment to Pets Among Owners of Cats and Dogs Using the Lexington Attachment to Pets Scale (LAPS) in the Basque Country
Source: Animals (Basel). 2025 Jan 1;15(1):76. doi: 10.3390/ani15010076 (PMC11718770; doi:10.3390/ani15010076)
Supplement: Supplementary file 1 [file animals-15-00076-s001.zip › Supplementary Table S1.pdf]

Supplementary Table 1: Lexington Attachment to Pets Scale: Spanish Version.

	Mean	SD	Corrected item-total correlation
**General attachment (α = 0.88, ω = 0.89)**			
10. Mi animal de compañia sabe cuando me siento mal	2.32	0.82	0.47
11. Muy seguido hablo de mi animal de compañia con otras personas	2.22	0.78219	0.50
12. Mi animal de compañia me entiende	1.94	0.87328	0.59
13. Pienso que el afecto que siento hacia mi animal de compañia me ayuda a estar saludable	2.38	0.81543	0.72
15. Mi animal de compañia y yo tenemos una relación muy cercana	2.53	0.74039	0.72
17. Juego con mi animal de compañia muy seguido	2.35	0.73992	0.58
18. Considero que mi animal de compañia es una magnífica compañía	2.69	0.62525	0.70
19. Mi animal de compañia me hace sentir feliz	2.67	0.63905	0.72
21. No estoy muy apegado a mi animal de compañía *(Reverse item)*	0.21	0.59787	-0.39
22. Tener un animal de compañia contribuye a mi felicidad	2.73	0.58830	0.61
23. Considero a mi animal de compañia un amigo	2.19	1.01597	0.64
**Substitution of people (α = 0.82, ω = 0.83)**			
1. Mi animal de compañia significa más para mí que cualquiera de mis amigos	1.70	1.02	0.70
2. Muy frecuentemente confío en mi animal de compañia	2.41	0.86	0.45
4. Creo que mi animal de compañia es mi mejor amigo	1.69	1.09	0.74
5. Es muy frecuente que mis sentimientos hacia la gente se vean afectados por como ellos reaccionan hacia mi animal de compañia	1.57	0.98	0.42
6. Quiero a mi animal de compañia porque es más leal conmigo que la mayoría de gente en mi vida	1.65	1.06	0.74
7. Disfruto mostrarle fotos de mi animal de compañia a la gente	2.11	0.95	0.43
9. Quiero a mi animal de compañia porque nunca me juzga	1.76	1.11	0.46
**Animal rights (α = 0.71, ω = 0.75)**			
3. Creo que los animales de compañia deben tener los mismos derechos y privilegios como miembros de la familia	2.11	0.97	0.17
8. Pienso que mi animal de compañia es solo una mascota *(Reverse item)*	1.19	1.10	-0.41
14. Los animales de compañia merecen tanto respeto como los humanos	2.74	0.59	0.39
16. Haría casi cualquier cosa por el cuidado de mi animal de compañia	2.56	0.71	0.35
20. Siento que mi animal de compañia es parte de la familia	2.76	0.54	0.49
