# Supplementary material for: A Study on the Attachment to Pets Among Owners of Cats and Dogs Using the Lexington Attachment to Pets Scale (LAPS) in the Basque Country
Source: Animals (Basel). 2025 Jan 1;15(1):76. doi: 10.3390/ani15010076 (PMC11718770; doi:10.3390/ani15010076)
Supplement: Supplementary file 1 [file animals-15-00076-s001.zip › Supplementary Table S2.pdf]

Supplementary Table 2. Lexington Attachment to Pets Scale (LAPS) results by pet owners and gender.

	Mean	SD	Median	Range
**LAPS Total score**				
**Cat owners**				
Men	42.4	12.71	44.5	11-62
Women	48.9	13.19	52	20-68
**Dog owners**				
Men	50.4	9.36	52	31-65
Women	55.3	10.81	59	16-69
**General Attachment**				
**Cat owners**				
Men	25.8	7.06	27	9-35
Women	28.3	7.03	31	9-36
**Dog owners**				
Men	29.5	4.74	31	16-36
Women	30.8	5.80	32	4-36
**Person Substitution**				
**Cat owners**				
Men	8.8	4.54	8.5	0-17
Women	11.5	5.05	12	2-20
**Dog owners**				
Men	11.9	3.94	15	4-19
Women	14.7	4.60	15	3-21
**Animal Rights**				
**Cat owners**				
Men	10.3	3.36	9.5	3-15
Women	11.7	2.98	12	3-15
**Dog owners**				
Men	11.7	2.71	12	6-15
Women	12.5	2.47	13	3-15
