# Supplementary material for: A Study on the Attachment to Pets Among Owners of Cats and Dogs Using the Lexington Attachment to Pets Scale (LAPS) in the Basque Country
Source: Animals (Basel). 2025 Jan 1;15(1):76. doi: 10.3390/ani15010076 (PMC11718770; doi:10.3390/ani15010076)
Supplement: Supplementary file 1 [file animals-15-00076-s001.zip › Supplementary Table S3.pdf]

Supplementary Table 3. Lexington Attachment to Pets Scale (LAPS) results by pet owners and children presence at home.

	Mean	SD	Median	Range
**LAPS Total score**				
**Cat owners**				
Living with children	46.2	13.36	49	23-68
Living without children	49.5	13.16	52	11-68
**Dog owners**				
Living with children	47.1	13.8	47	16-69
Living without children	55.8	9.25	57	21-69
**General Attachment**				
**Cat owners**				
Living with children	27.5	6.56	29	17-36
Living without children	28.8	6.83	31	9-36
**Dog owners**				
Living with children	27.4	8.19	29	4-36
Living without children	31.3	4.66	32	7-36
**Person Substitution**				
**Cat owners**				
Living with children	10.4	5.02	9	4-20
Living without children	11.5	5.01	12	0-20
**Dog owners**				
Living with children	11.5	4.64	12	3-21
Living without children	14.6	4.35	15	3-21
**Animal Rights**				
**Cat owners**				
Living with children	10.8	3.45	12	3-15
Living without children	10.9	2.97	13	3-15
**Dog owners**				
Living with children	10.6	3.15	11	3-15
Living without children	12.7	2.15	13	7-15
